# Supplementary material for: Integrated serosurveillance to assess disease elimination in coastal Ecuador: onchocerciasis, yaws, and trachoma
Source: medRxiv. 2026 Feb 19:2026.02.16.26346420. Preprint. [Version 2] doi: 10.64898/2026.02.16.26346420 (PMC12934873; doi:10.64898/2026.02.16.26346420)
Supplement: 1 [file NIHPP2026.02.16.26346420V2-supplement-1.pdf]

medRxiv preprint doi: https://doi.org/10.64898/2026.02.16.26346420; this version posted February 19, 2026. The copyright holder for this preprint (which was not certified by peer review) is the author/funder, who has granted medRxiv a license to display the preprint in perpetuity.
It is made available under a CC-BY 4.0 International license .

Integrated serosurveillance to assess disease elimination in coastal Ecuador: onchocerciasis, yaws, and trachoma

Simbaña et al.

Supplementary Information Materials

medRxiv preprint doi: https://doi.org/10.64898/2026.02.16.26346420; this version posted February 19, 2026. The copyright holder for this preprint (which was not certified by peer review) is the author/funder, who has granted medRxiv a license to display the preprint in perpetuity.

It is made available under a CC-BY 4.0 International license .

Supplementary Table 1. Cumulative incidence of *Chlamydia trachomatis* IgG seroconversion between ages 6 and 24 months in Esmeraldas Province, Ecuador 2021-2024. Esmeraldas is an urban environment, Borbon is intermediate urbanicity, and Rural villages include eight communities accessed by road or river.

Population	N At Risk	Incident seroconversions	Cumulative Incidence (95% CI)*
**Overall**	352	37	10.5 (7.5 to 14.2)
**Location**			
Esmeraldas city	85	3	3.6 (0.8 to 10.2)
Borbon	108	2	1.9 (0.2 to 6.8)
Rural villages	177	32	19.4 (13.7 to 26.3)

*Cumulative incidence per 100 children at risk. 95% confidence intervals estimated using an exact binomial distribution.

Supplementary Table 2. Seroprevalence to *Chlamydia trachomatis* Pgp3 IgG at age 6 months in Esmeraldas province, Ecuador, 2021-2024. Esmeraldas is an urban environment, Borbon is intermediate urbanicity, and Rural villages include eight communities accessed by road or river.

Population	N children	N positive	Seroprevalence (95% CI)*
**Overall**	370	18	4.9 (2.9 to 7.6)
**Birth type**			
Cesarean section	63	0	0.0 (0.0 to 5.7)
Vaginal delivery	307	18	5.9 (3.5 to 9.1)
**Location**			
Esmeraldas city	85	2	2.4 (0.3 to 8.2)
Borbon	108	4	3.7 (1.0 to 9.2)
Rural villages	177	12	6.8 (3.6 to 11.5)

* 95% confidence intervals estimated using an exact binomial distribution.

medRxiv preprint doi: https://doi.org/10.64898/2026.02.16.26346420; this version posted February 19, 2026. The copyright holder for this preprint (which was not certified by peer review) is the author/funder, who has granted medRxiv a license to display the preprint in perpetuity.
It is made available under a CC-BY 4.0 International license.

Supplementary Table 3. *Chlamydia trachomatis* Pgp3 IgG seroreversion rates (SRR) estimated longitudinally among children ages 6-24 months in Esmeraldas Province, Ecuador 2021-2024. Rates were estimated over the whole age range, 6-24 months, and restricted to 12-24 months to avoid potential for seroreversion due to waning maternal IgG.

Age range	N children	Incident sero-reversions	Person-years at risk	SRR (95% CI) *
6 to 24 months	370	26	23.3	111.4 (68.8 to 180.8)
12 to 24 months	352	13	14.1	92.1 (46.4 to 160.2)

* SRR: seroreversion rate per 100 person-years. SRR was estimated longitudinally with bootstrap 95% confidence intervals that resampled children with replacement.

medRxiv preprint doi: https://doi.org/10.64898/2026.02.16.26346420; this version posted February 19, 2026. The copyright holder for this preprint (which was not certified by peer review) is the author/funder, who has granted medRxiv a license to display the preprint in perpetuity.

It is made available under a CC-BY 4.0 International license .

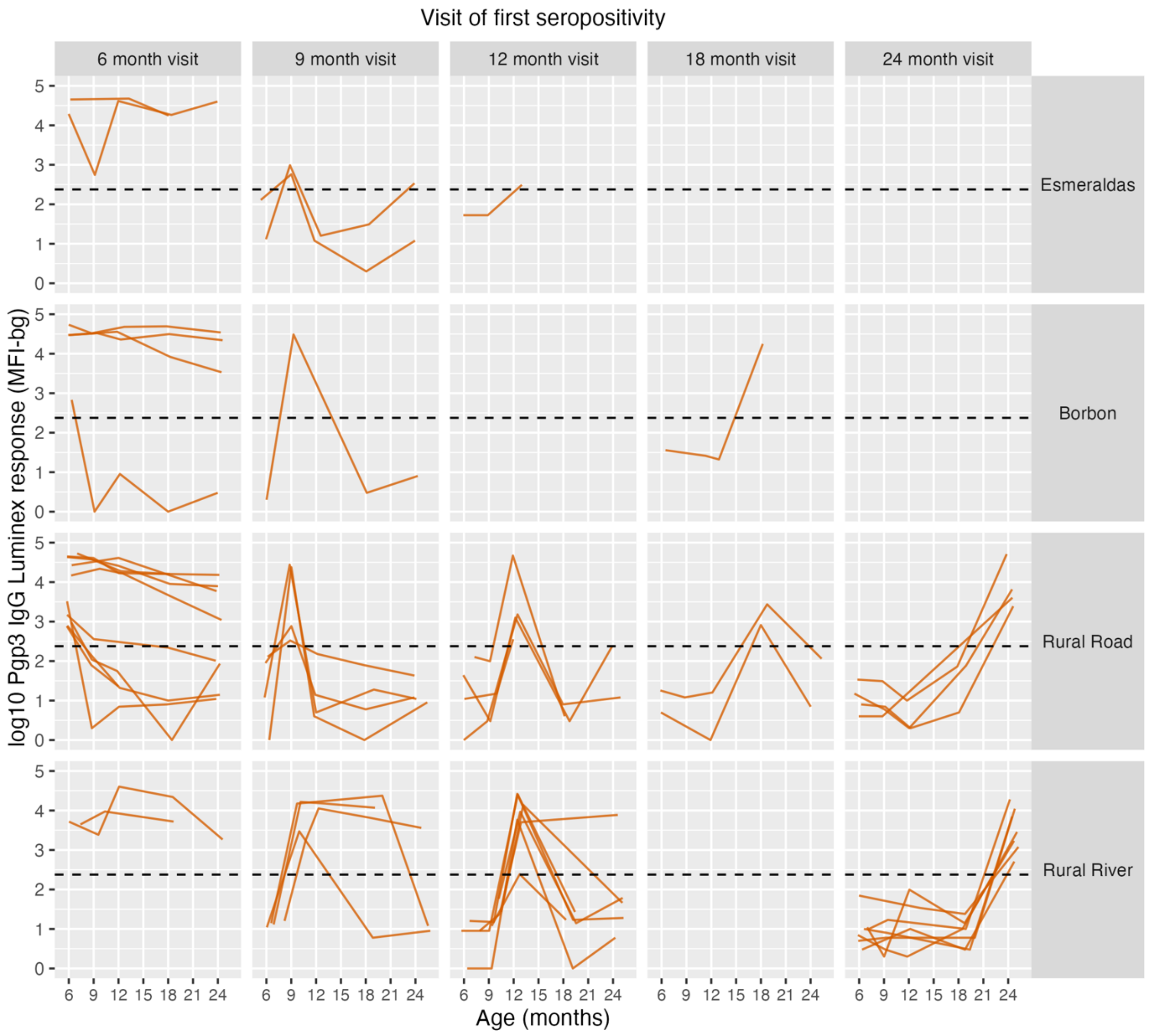

Supplementary Figure 1. Longitudinal trajectories of *Chlamydia trachomatis* IgG responses to Pgp3 antigen among 55 children in the cohort who were seropositive during follow-up between ages 6 and 24 months in Esmeraldas, Ecuador, 2021-2024. Children are stratified by the visit at which they were first identified as seropositive to Pgp3 and by community type. IgG measured in Median Florescence Units minus background (MFI-bg) on the Luminex platform. A horizontal dashed line marks the seropositivity cutoff.

medRxiv preprint doi: https://doi.org/10.64898/2026.02.16.26346420; this version posted February 19, 2026. The copyright holder for this preprint (which was not certified by peer review) is the author/funder, who has granted medRxiv a license to display the preprint in perpetuity.
It is made available under a CC-BY 4.0 International license .

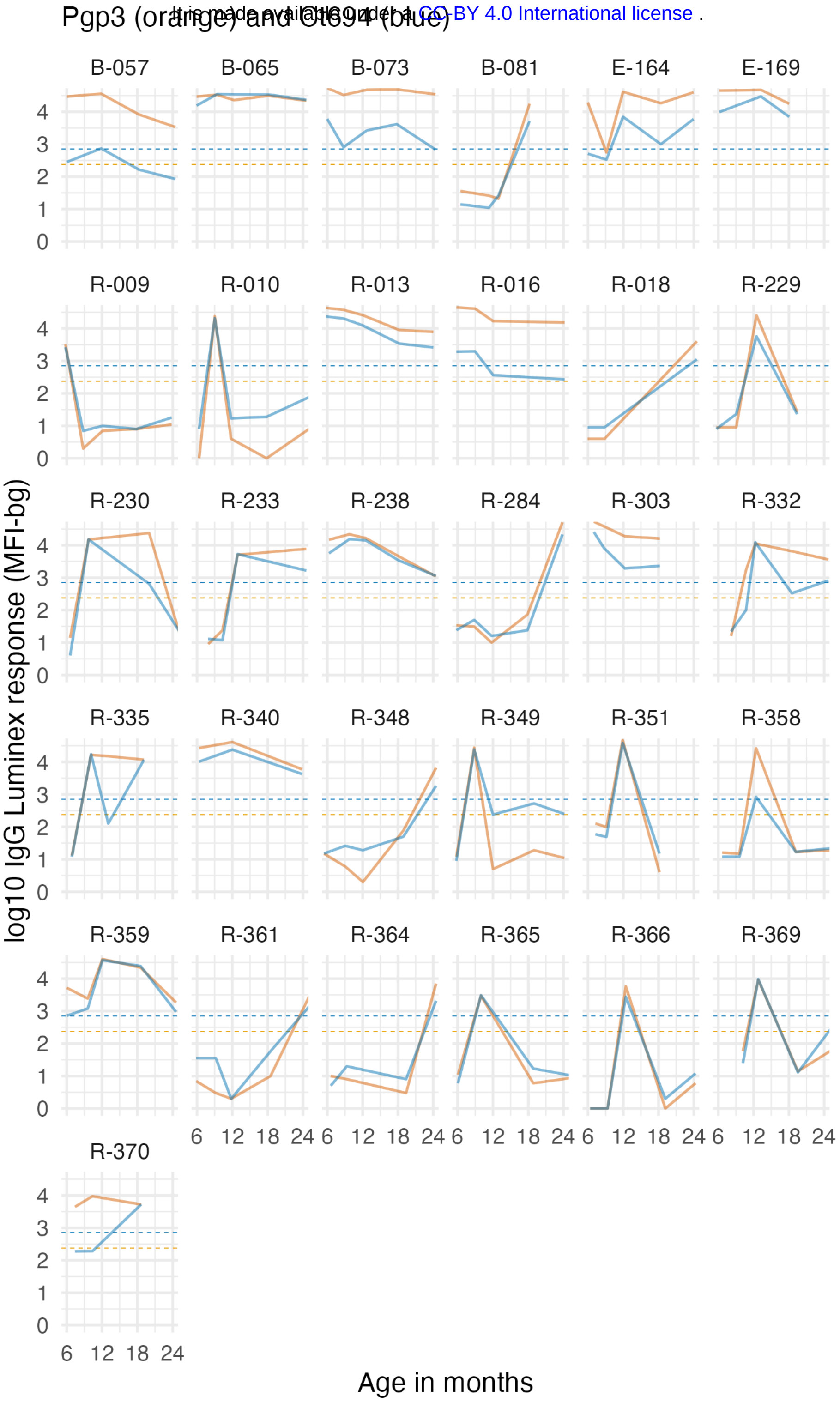

Supplementary Figure 2. Longitudinal trajectories of *Chlamydia trachomatis* IgG responses to Pgp3 and Ct694 antigens among 31 children in the cohort who were seropositive to both antigens during follow-up between ages 6 and 24 months in Esmeraldas, Ecuador, 2021-2024. IgG measured in Median Florescence Units minus background (MFI-bg) on the Luminex platform. Pgp3 IgG levels are shown in orange and Ct694 IgG levels are shown in blue. Dashed lines indicate seropositivity cutoffs for each antigen. De-identified child IDs in each panel identify their community group: Esmeraldas city (E), Borbón (B), and Rural (R) villages.

medRxiv preprint doi: https://doi.org/10.64898/2026.02.16.26346420; this version posted February 19, 2026. The copyright holder for this preprint (which was not certified by peer review) is the author/funder, who has granted medRxiv a license to display the preprint in perpetuity.

It is made available under a CC-BY 4.0 International license .

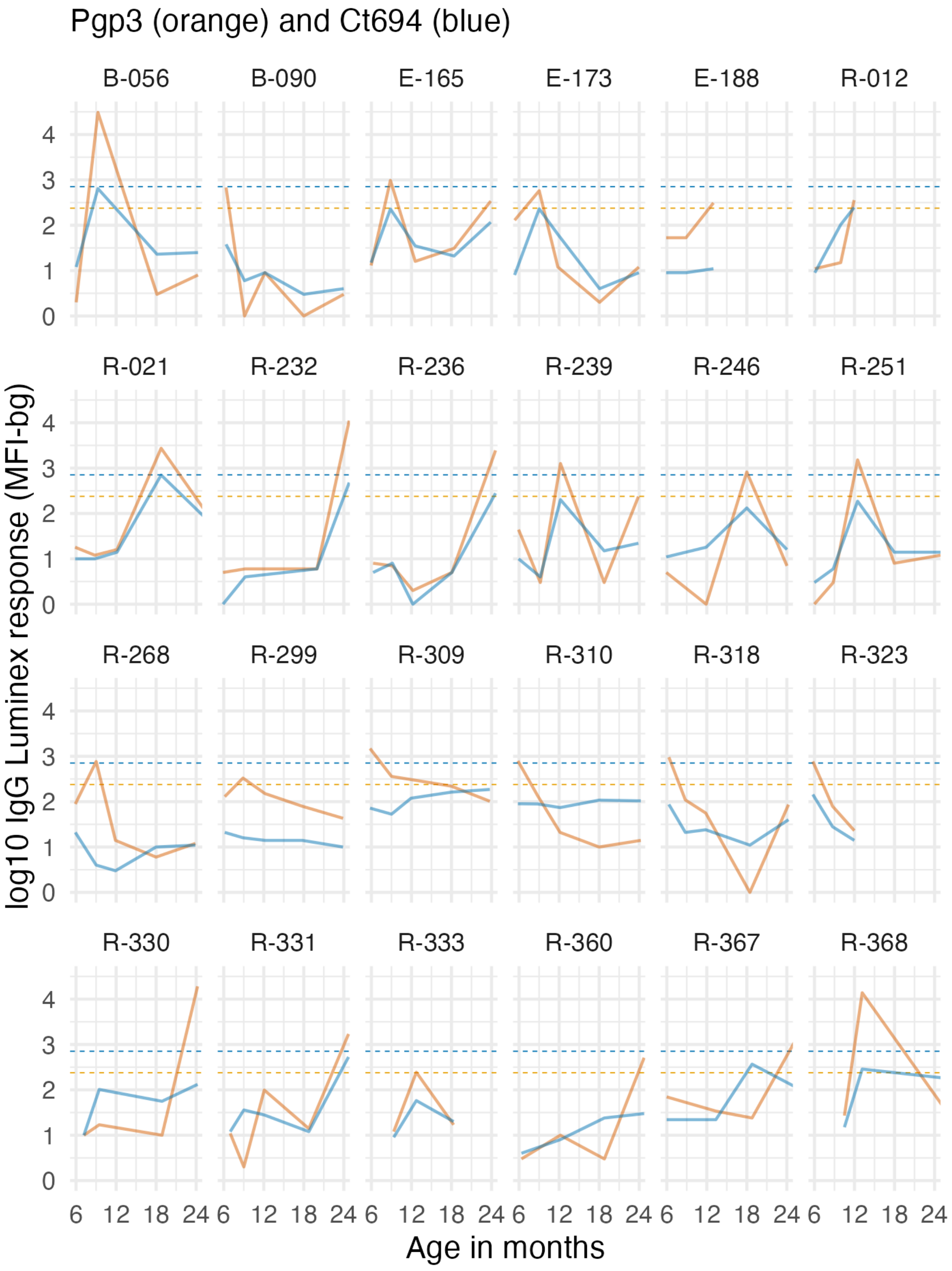

Supplementary Figure 3. Longitudinal trajectories of *Chlamydia trachomatis* IgG responses to Pgp3 and Ct694 antigens among 24 children in the cohort who were seropositive to Pgp3 alone (not Ct694) during follow-up between ages 6 and 24 months in Esmeraldas, Ecuador, 2021-2024. IgG measured in Median Florescence Units minus background (MFI-bg) on the Luminex platform. Pgp3 IgG levels are shown in orange and Ct694 IgG levels are shown in blue. Dashed lines indicate seropositivity cutoffs for each antigen. De-identified child IDs in each panel identify their community group: Esmeraldas city (E), Borbón (B), and Rural (R) villages.
